# From guidelines to evidence-based practice – A German perspective on mesalazine as first-line therapy for mild-to-moderate ulcerative colitis

**DOI:** 10.1055/a-2596-8934

**Published:** 2025-06-16

**Authors:** Elisabeth Schnoy, Axel Dignass, Matthew Gaskins, Torsten Kucharzik

**Affiliations:** 1 39694 Internal Medicine III, University Hospital Augsburg, Augsburg, Germany; 2 84491 Department of Medicine I, Goethe-University, Agaplesion Markus Hospital, Frankfurt, Germany; 3 Independent public health researcher, Madrid, Spain; 4 Department of Gastroenterology, Lüneburg Hospital, Lüneburg, Germany

**Keywords:** 5-aminosalicylic acid, evidence-based practice, guidelines, mesalamine, mesalazine, mild-to-moderate UC, ulcerative colitis, Colitis ulcerosa, 5-Aminosalicylsäure, evidenzbasierte Praxis, Leitlinien, Mesalamin, Mesalazin, leichte bis mäßiggradige Colitis ulcerosa, chronisch entzündliche Darmerkrankung

## Abstract

Mesalazine is the first-line treatment for mild-to-moderate ulcerative colitis (UC) of any extent, as recommended by all major international and national guidelines. Approximately 85% of UC cases are classified as mild-to-moderate, making mesalazine a cornerstone therapy for the majority of patients. It rapidly induces clinical response and clinical remission, sustains steroid-free clinical, endoscopic, and histologic remission over the long term, and has a safety profile comparable to placebo. This paper reviews the recommendations for mesalazine use in the German UC guideline and provides practical advice (including do’s and don’ts) for their implementation in daily clinical practice. Examples include explaining the expected timeline and nature of the clinical response to mesalazine treatment; outlining the practical implications of the dose-dependency of the drug’s therapeutic effect; and emphasizing the importance of rectal mesalazine as the first-line treatment for proctitis. Additionally, we conducted a systematic literature search to evaluate whether mesalazine should be continued after escalation to biologics or small molecules. While no clear evidence of short-term clinical benefit was found, there was also no evidence of harm. In light of the potential long-term chemoprotective effect of mesalazine, continuation may be considered on a case-by-case basis. Lastly, we provide an overview of the various mesalazine formulations available in Germany, detailing how they are not interchangeable due to differences in drug-release profiles, excipients, and dosing strengths. Understanding these differences may help clinicians personalize treatment, improving adherence and clinical outcomes.

## Zusammenfassung

Mesalazin wird in allen bedeutenden internationalen und nationalen Leitlinien als Erstlinienbehandlung bei leicht- bis mäßiggradiger Colitis ulcerosa (CU) jeglicher Ausdehnung empfohlen. Da ungefähr 85% der CU-Fälle als leicht bis mäßiggradig eingestuft werden, stellt Mesalazin die Basistherapie für den Großteil der Patient/-innen dar. Es führt rasch zu einem klinischen Ansprechen und einer klinischen Remission, erhält langfristig eine steroidfreie klinische, endoskopische und histologische Remission aufrecht und weist ein mit Placebo vergleichbares Sicherheitsprofil auf. In diesem Artikel werden die Empfehlungen für die Anwendung von Mesalazin in der deutschen CU-Leitlinie diskutiert und praktische Tipps (einschließlich Do’s und Don’ts) für deren Umsetzung im klinischen Alltag gegeben. Beispiele hierfür sind eine Erläuterung der zu erwartenden Dauer bis zum klinischen Ansprechen sowie des klinischen Verlaufs unter Mesalazin-Therapie, die Darstellung der praktischen Implikationen der dosisabhängigen therapeutischen Wirkung des Medikaments und die Betonung der wichtigen Rolle von rektalem Mesalazin als Erstlinientherapie bei Proktitis. Darüber hinaus wurde eine systematische Literaturrecherche durchgeführt, um zu bewerten, ob Mesalazin nach einer Eskalation auf Biologika oder small molecules fortgesetzt werden sollte. Zwar konnte keine eindeutige Evidenz für einen kurzfristigen klinischen Nutzen gefunden werden, jedoch auch keine Hinweise auf einen Schaden. Vor dem Hintergrund der potenziellen langfristigen chemoprotektiven Wirkung von Mesalazin kann eine Fortführung der Therapie im Einzelfall in Erwägung gezogen werden. Abschließend wird ein Überblick über die verschiedenen in Deutschland erhältlichen Mesalazin-Formulierungen gegeben und erläutert, warum sie aufgrund unterschiedlicher Wirkstofffreisetzungsprofile, Hilfsstoffe und Dosierungsstärken nicht austauschbar sind. Das Verständnis dieser Unterschiede kann Ärztinnen und Ärzte dabei helfen, die Behandlung individuell anzupassen und so die Therapietreue und die klinischen Ergebnisse zu verbessern.

## Introduction

Ulcerative colitis (UC) is a chronic inflammatory bowel disease (IBD) characterized by mucosal inflammation that extends in a continuous manner from the rectum through various lengths of the colon. Individuals with UC commonly experience alternating phases of spontaneous flares, drug-induced remission, and varying degrees of chronic disease activity 1 2 3 . The disease is associated with debilitating physical symptoms, such as bloody diarrhea, urgency, fecal incontinence, and abdominal pain, as well as psychological distress, which can substantially impair health-related quality of life (QoL) and general life satisfaction 3 4 . Even during periods of clinical remission, inflammation may continue, leading to persistent urgency, IBS-like symptoms, post-inflammatory complications including neoplasia, and extraintestinal manifestations, such as peripheral arthritis, primary sclerosing cholangitis, and pyoderma gangrenosum, which can occur in approximately one-third of patients 5 .

The global burden of UC is substantial and continues to grow, with an estimated five to seven million people affected worldwide in 2023 6 7 8 . This increasing prevalence is driven by multiple factors, including environmental changes 9 and lifestyle adaptations 10 in both high- and low-income countries. In the former, the growing number of cases among older individuals and demographic aging pose major challenges, with age-associated changes in the immune system, sarcopenia, and comorbidities adding complexity to UC management. The burden of UC in Germany is also considerable, with a recent analysis of health insurance claims data indicating a prevalence surpassing 0.5% 11 . The etiology of UC remains elusive, although a range of factors including genetic predisposition, environmental influences, luminal factors, and mucosal immune dysregulation have been demonstrated to contribute to its pathogenesis 12 .

In the absence of a cure for UC, the primary therapeutic goals in treatment have evolved from providing symptomatic relief to achieving and sustaining steroid-free mucosal healing with well-tolerated medication. This approach aims to avoid surgical interventions, reduce the use of systemic steroids, decrease the likelihood of colorectal cancer, and improve quality of life 13 14 . Since the 1950s and continuing to this day, the approach to managing UC has been structured around a step-up treatment model, with 5-aminosalicylic acid (5-ASA) therapies playing a central role. This class includes sulfasalazine, olsalazine, balsalazide, and mesalazine (also known as mesalamine). Among these, mesalazine has become the most widely used and extensively studied due to its improved safety profile and targeted drug delivery 14 15 16 17 . The current manuscript will focus exclusively on mesalazine.

The most recent national and international guidelines, including those from the European Crohn’s and Colitis Organisation (ECCO) 16 , the German Society for Gastroenterology, Digestive and Metabolic Diseases (DGVS) 18 , the American College of Gastroenterology (ACG) 14 , and the British Society of Gastroenterology (BSG) 19 , consistently recommend mesalazine as the first-line treatment of choice for patients with mild-to-moderate UC. Mesalazine is also recommended as first-line therapy for these patients in a recent international expert consensus statement, which additionally points to the development of new ambitious outcomes such as histological remission and disease clearance 20 . While guideline development groups are continually assessing the role of mesalazine as the treatment landscape broadens to include more biologics and small molecules, the most recent guidelines still do not recommend early intervention with these newer therapies in mild-to-moderate cases. Instead, they suggest tailoring the dose of mesalazine to the severity and phase of the disease, starting with higher doses that are maintained long enough to reduce the risk of relapse 14 15 16 18 21 . This restraint is based not only on the scarcity of evidence on comparative effectiveness but also on the well-established high effectiveness of mesalazine and its favorable safety profile. Given that over 85% of UC patients have mild-to-moderate disease 22 , mesalazine remains the cornerstone of UC treatment.

The primary aim of this manuscript is to review the recommendations for mesalazine use in the most recent German clinical practice guideline 18 , clarify why mesalazine continues to be the first-line treatment of choice, and provide expert, practical advice for translating these recommendations into daily, evidence-based clinical practice. We will also conduct a systematic search of the literature to identify evidence on whether and how to continue mesalazine treatment alongside newer therapies. The secondary aim is to provide an overview of the many mesalazine products currently on the market in Germany, including their drug release profiles, dosage forms, and strengths in order to support patient-individualized mesalazine therapies.

Our overview and findings will be useful to IBD specialists seeking to optimize the effectiveness and efficiency of UC treatment by highlighting the differences between the various mesalazine products and their distinct characteristics.

## Methods

This paper provides a narrative review of the recommendations for mesalazine use within the most recent German clinical practice guideline for the treatment of mild-to-moderate UC, integrating expert opinion and practical advice from the authors for their implementation in everyday clinical practice. Insights and practical recommendations are based on consensus among the authors and are informed by their professional expertise and targeted searches of the literature.

Importantly, the German guideline recommendations presented in this paper are in condensed form. The original guideline contains more detailed wording, including gradations in the strength of recommendations and evidence levels, which are essential for making informed treatment decisions. For precise details and to ensure comprehensive understanding, readers are encouraged to consult the full text of the guideline 18 . The summaries presented here are intended to provide an accessible overview and should not replace consultation of the detailed guideline document.

Additionally, we conducted a systematic literature search in PubMed in January 2025, using a pre-defined search string and inclusion/exclusion criteria. The search examined existing evidence on the potential benefits and harms of continuing mesalazine therapy after escalation to biologics or small molecules in patients with ulcerative colitis (UC). Detailed descriptions of the search strategy, including the search string, research question formatted using the PICO (Population, Intervention, Comparison, Outcome) framework, and the inclusion and exclusion criteria are provided in the Supplementary Appendix. The resulting records were independently reviewed by two of the authors at the title/abstract and full-text stages, with disagreements resolved by consensus. The search included randomized controlled trials (RCTs), controlled clinical trials, observational studies, retrospective and prospective cohort studies, and analyses of clinical trial data, with eligibility limited to peer-reviewed papers published in English between January 1, 1995, and January 29, 2025.

Detailed summaries of the included studies, their relevance to the research question, and a narrative synthesis of the search results are also provided in the Supplementary Appendix.

## Continuing role of mesalazine as first-line therapy in mild-to-moderate UC

**Fig. 1:**
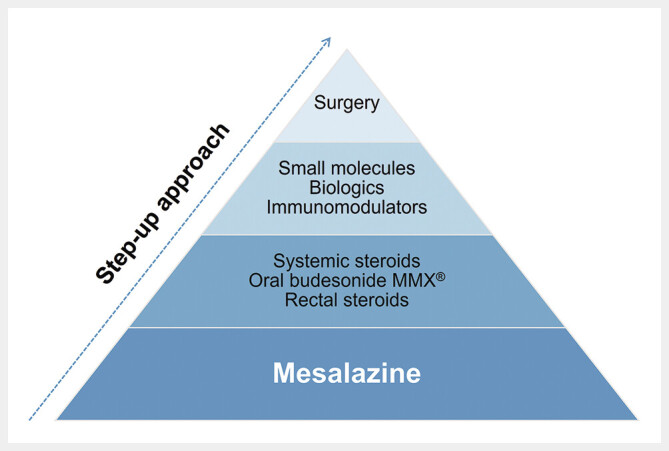
Treatment pyramid for ulcerative colitis 16 18 23 24 .

The management of mild-to-moderate UC has relied on a step-up approach, in which decisions to intensify treatment are guided by clinical symptoms, including non-response or intolerance to initial therapies. This approach can be visualized as a treatment pyramid ( Fig. 1 ), with mesalazine (oral and rectal, both as mono and combination therapies) being the recommended first-line treatment at the base of the pyramid, followed by escalation to low-bioavailability/colonic-release corticosteroids, systemic steroids, immunosuppressants, and biologics/small molecules, with surgery at the tip 16 18 23 24 .

While mesalazine has always played a central role in this step-up approach, the advent of biologics and small molecule therapies has broadened the range of treatment options available. This has led to discussions about the potential benefits of top-down strategies, which would involve starting treatment with more aggressive drugs. The rationale for top-down therapy – which has been discussed more extensively in patients with Crohn’s disease 16 – is to induce remission more quickly. However, as we will outline in the following sections, substantial evidence shows that mesalazine can rapidly induce clinical remission within as little as two weeks, followed by lasting steroid-free clinical, endoscopic, and histologic remission. Indeed, unlike in Crohn’s disease, there is less evidence that early initiation or early treatment escalation with these newer therapies has any benefit for patients with mild-to-moderate UC 25 .

Additionally, mesalazine has been shown to have a safety profile comparable to that of placebo 16 23 24 , whereas most biologics and small molecule therapies come with more substantial risks. These include an increased risk of infections, infusion and hypersensitivity reactions, a slightly increased risk of certain cancers for some therapies and, in the case of JAK inhibitors, risks such as blood clots, elevated liver enzyme, and increased cholesterol levels 26 27 28 29 30 . Mesalazine therefore continues to be recommended as the first-line therapy of choice for inducing and maintaining remission in mild-to-moderate UC across all of the major UC guidelines 14 15 16 18 21 , including the most recent update of the German Guideline on Ulcerative Colitis.

### German guideline recommendations and advice for everyday clinical practice

#### Induction of remission in mildly to moderately active proctitis

**Table:** **Table 1Box 1** . Induction treatment of mildly to moderately active proctitis

* Summarized recommendations (and related reference numbers from the guideline) ^‡^*	
3.5	Treat proctitis initially with rectal mesalazine ≥1 g/day using suppositories; alternatively, use rectal foams or enemas.
3.6	If rectal monotherapy with mesalazine fails, combine it either with rectal steroids or oral mesalazine.
^‡^ Adapted from Kucharzik et al., 2024 18 . Please note that the information presented here and in all subsequent summary boxes represents a condensed version of the recommendations in the German UC guideline. The full wording of the recommendations in the UC guideline considers gradations in recommendation and evidence strength and should be consulted prior to making any treatment decisions	

For proctitis ( **Box 1** ), the German guideline recommends starting with rectal therapy using mesalazine suppositories at a dose of at least 1 g/day. Mesalazine foam or enemas can be used instead, but these are described as alternatives because they may not be as well tolerated by patients. If treatment with rectal mesalazine fails, the guideline recommends that it should be combined with either topical steroid therapy or oral mesalazine-releasing preparations (≥3g/day). In cases where oral mesalazine is used as add-on treatment, the guideline notes that once-daily dosing with mesalazine granules in particular appears to be beneficial. If this approach also fails, the guideline advises confirming therapy adherence and endoscopic findings, and, if necessary, applying the principles used to treat severe ulcerative colitis of any extent 18 .

#### Translating the recommendations into evidence-based clinical practice

*In patients with proctitis only, the guideline recommends initiating treatment with rectal mesalazine suppositories. However, oral therapy alone is commonly prescribed. Why is this the case, and what is the optimal approach?*

Rectal mesalazine is strongly recommended as the first-line treatment for proctitis because it delivers high concentrations of the active drug directly to the site of inflammation, offering superior effectiveness to oral therapy alone. However, local therapy may be underutilized due to several factors, including clinician familiarity and comfort with oral regimens, patient reluctance or discomfort with rectal administration, and potential time constraints in fully explaining rectal therapy options during consultations. To address this, clinicians should actively consider and discuss rectal mesalazine as a primary treatment option for proctitis, emphasizing its effectiveness and generally favorable tolerability. Providing clear instructions and reassurance about the use of suppositories, foams, or enemas can help increase patient acceptance.

*What advice should I give to my patients on how best to administer rectal mesalazine?*

Proper application techniques are crucial: suppositories should be inserted blunt end first after warming by hand and moistening with water 31 . Foams should not be expelled prematurely but should be retained for at least 10–15 seconds after application. Mesalazine enemas should be applied while patients are lying on their left lateral side, and they should maintain this position for about 30 minutes to ensure maximum absorption.

*How and when should treatment failure with initial rectal mesalazine be determined?*

The guideline does not specify the exact signs or duration for determining when initial treatment of proctitis with rectal mesalazine has failed. However, clinical practice generally considers a lack of symptom improvement or worsening of symptoms within two to four weeks as indicative of treatment failure. Persistent bleeding, ongoing inflammation, and patient-reported outcomes are key indicators to assess the effectiveness of the therapy. In cases where patients are unable to retain a rectal enema for at least 30 minutes, suppositories may be used as an alternative and may be better tolerated. If these options are not effective or feasible, it may indicate severe disease requiring escalation to topical or systemic corticosteroids or other treatments.

*What is the best combination treatment and dosage if initial rectal mesalazine for proctitis fails?*

The guideline does not explicitly state which combination should be preferred. However, clinical practice suggests that the choice often depends on patient preference. Adding oral mesalazine is often preferred due to its broader reach within the digestive tract, which can address inflammation beyond the rectum. However, some patients also prefer rectal treatment only and combine topical steroids and rectal mesalazine, using one drug in the morning and the other in the evening. Topical steroids can be effective but may be reserved for patients who do not respond adequately to combined oral and rectal mesalazine therapy, bearing in mind that long-term use of systemic steroids should be avoided due to side effects.

The guideline does not provide a specific dosage for adding oral mesalazine when rectal mesalazine treatment is insufficient. In German clinical practice, doses of 2 to 4.8 g/day tend to be used to achieve optimal therapeutic effects, often depending on which mesalazine formulation is chosen (see also Section 4 “Overview of mesalazines and differences”). The exact dosage may be adjusted based on the mesalazine formulation used, as well as the patient’s response, tolerance, and disease severity.

### Induction of remission in mildly to moderately active UC of at least rectosigmoid extent

This category encompasses all forms of active UC that extend proximally beyond the rectum. It thus includes proctitis (E1 according to the Montreal classification), left-sided colitis (E2), and extensive colitis (also known as pancolitis, E3). Importantly, the German guideline divides its recommendations in this category into two subcategories: those for “left-sided colitis” and for “extensive colitis”. The term “left-sided colitis” is used to refer to disease extending up to the splenic flexure 18 32 , and thus the recommendations in this subcategory also apply to proctosigmoiditis.

### Mildly to moderately active left-sided colitis

**Table:** **Table 2Box 2** . Induction treatment of mildly to moderately active left-sided colitis

* Summarized recommendations (and related reference numbers from the guideline) ^‡^*	
3.7	Treat initially with rectal mesalazine (≥1 g/day, enema or foam) in combination with oral mesalazine (≥3 g/day).
3.8	Prefer rectal mesalazine therapy over topical steroid therapy.
3.9	Prefer daily single-dose administration of oral mesalazine.
^‡^ Adapted from Kucharzik et al., 2024 18 .	

For induction treatment of mildly to moderately active left-sided colitis ( **Box 2** ), the German guideline recommends a combination of rectal mesalazine (≥ 1 g/day, enema or foam) and oral mesalazine (≥3 g/day) for initial treatment. The guideline explains that this combination is favored over oral mesalazine monotherapy due to its higher response rates and faster onset of action, pointing to a systematic review and meta-analysis from 2012 33 . In the event of intolerance to rectal application, however, the guideline notes that oral monotherapy may be considered. The guideline recommends that rectal mesalazine should be preferred over topical steroid therapy.

The guideline also underscores the crucial role of treatment adherence in mesalazine therapy. It encourages physicians to discuss dosing options and formulations with patients, such as once-daily dosing and the choice between tablets or granules. In doing so, daily single-dose administration of oral mesalazine is recommended due to its equivalence in effectiveness to multiple daily doses, with the added benefit of simplifying the treatment regimen, which may improve adherence. Moreover, the guideline emphasizes the necessity of administering sufficiently high doses of mesalazine (at least 3 g/day) for all patients with mild-to-moderate disease, citing evidence that patients with moderate disease may benefit from a higher initial dose of 4.8 g/day 34 .

### Extensive mildly to moderately active UC

**Table:** **Table 3Box 3** . Induction treatment of extensive mildly to moderately active UC

* Summarized recommendation (and related reference number from the guideline) ^‡^*	
3.11	Treat initially with oral mesalazine (≥3 g/day) in combination with rectal mesalazine treatments such as enemas or foams.
^‡^ Adapted from Kucharzik et al., 2024 18 .	

For induction treatment of extensive mildly to moderately active UC ( **Box 3** ), the recommendation is essentially the same as that for left-sided colitis. As with left-sided colitis, the guideline points to evidence of the superior efficacy of combining oral mesalazine with rectal therapy compared to oral monotherapy. Once again, the guideline explicitly emphasizes the importance of ensuring adequate dosing of mesalazine, of at least 3 g/day.

#### Translating the recommendations into evidence-based clinical practice

*Is the therapeutic effect of mesalazine dose-dependent? What about side effects?*

Yes, the therapeutic effect of mesalazine appears to be dose-dependent. Higher doses of mesalazine (>3 g/day) have been shown to be more effective in inducing remission in patients with mild-to-moderate UC. A 2018 systematic review and network meta-analysis by Nguyen et al. found that while standard-dose mesalazine (2–3 g/day) is usually sufficient for inducing remission in mild UC, higher doses are preferable for moderate cases 35 . A more recent network meta-analysis conducted by Barberio et al. (2021) involving 11,733 patients, showed that higher doses of oral mesalazine (≥3.3 g/day) were significantly more efficacious than lower doses for sustainable induction of remission 36 . Among the 20 included RCTs that used high-dose oral mesalazine, nine used 4.8 g/day, one 4.5 g/day, six 4 g/day and four 3.6 g/day 36 . This evidence supports the use of higher doses during the induction phase to maximize the chances of achieving remission, aligning with the German guideline’s recommendation for higher induction doses of 3 g/day or greater. In contrast, the side effects of mesalazine therapy are **not** dose-dependent 37 38 and have been shown to be similar to placebo 26 38 39 40 .

*How quickly and what kind of response should be expected from mesalazine treatment?*

The German guideline does not explicitly define the duration of a rapid response. Achieving rapid clinical response in UC is crucial and should be considered an immediate treatment target because symptomatic relief is highly prioritized by patients, as emphasized by the STRIDE-II statement 41 . According to STRIDE-II, physicians should consider changing treatment if there is no significant improvement within approximately four weeks or no improvement at all after two weeks when using mesalazine. Studies investigating different oral mesalazine products at higher daily doses (4 g/day or 4.8 g/day), with or without rectal mesalazine, suggest that nearly half of patients with mild-to-moderate UC may be able to achieve symptom resolution within two weeks 42 , and as many as one-third may reach clinical remission within the same period 43 . The expected improvements in symptoms indicating a rapid clinical response to mesalazine include reduced bowel movement frequency, less bleeding, and decreased abdominal pain 41 .

*Can first-line treatment with mesalazine be optimized to avoid treatment failure?*

Optimization of mesalazine treatment generally involves maximizing oral doses of the drug, combining oral with rectal therapies to improve effectiveness and potentially avoid the need for steroids or other escalating therapies, as well as opting for simple once-daily treatment 23 . Several studies have demonstrated that using optimization approaches from the beginning of therapy can help prevent the progression of disease, avoid the need for therapy escalation, reduce side effects, and save costs 28 44 .

Incorporating a treat-to-target strategy into clinical practice can further optimize mesalazine use. The recent OPTIMISE study underscores the value of guiding treatment strategies using measurable targets such as mucosal healing rather than focusing only on symptomatic relief 45 . This approach not only aims to achieve deeper remission states but also facilitates personalized, dynamic therapy adjustments based on non-invasive markers such as fecal calprotectin combined with clinical symptoms (PRO-2) 45 .

*In patients who respond well to induction therapy with mesalazine, how long should induction therapy be continued?*

The German guideline does not specify the exact duration of induction therapy with mesalazine in patients with mild-to-moderate UC. However, evidence from a recent non-interventional prospective Dutch study 46 suggests that a longer duration of induction therapy (>6 months) is associated with a substantially reduced risk of recurrence, particularly among patients receiving higher (≥4 g/day) doses of mesalazine. These findings confirm earlier similar findings supporting a duration of at least three months 47 . In summary, if patients are responding to therapy, continuing the recommended induction dose of mesalazine for six months or more is a reasonable, evidence-based approach, particularly given the favorable safety profile of mesalazine 26 38 39 40 .

*When and how should mesalazine therapy be de-escalated?*

De-escalation refers to reducing the intensity or dosage of therapy once remission is achieved. After the initial induction period and once clinical remission is achieved, the higher dose of mesalazine is generally continued for an additional period to stabilize remission and achieve at least endoscopic remission or even complete deep healing (i.e., clinical remission plus complete endoscopic and histological healing) 41 , with evidence supporting a total duration of induction therapy of at least six months 46 . After this period, the dose can be reduced to a maintenance level (≥2 g/day) to sustain remission. Further de-escalation is not recommended by the German guideline, which explicitly recommends offering patients long-term treatment with ≥2 g/day of mesalazine due to its potential cancer protection effects and favorable safety profile. We will discuss these topics more below in the section on maintenance and long-term mesalazine therapy.

### Escalating mesalazine induction treatment

**Table:** **Table 4Box 4** . Escalating mesalazine induction treatment

* Summarized recommendations (and related reference numbers from the guideline) ^‡^*	
3.10a/3.12	Use systemic steroid therapy (0.5–1 mg/kg body weight/day of prednisolone equivalent) if the approaches recommended in 3.5–3.9 and 3.11 fail or if a severe form of UC is already present at diagnosis.
3.10b	Budesonide MMX 9mg/day is a recommended option in mild-to-moderate left-sided colitis if the patient does not respond to or tolerate mesalazine.
^‡^ Adapted from Kucharzik et al., 2024 18 .	

If the recommended first-line induction treatments for mildly to moderately active left-sided colitis or extensive mildly to moderately active UC fail, or if a severe form of UC is already present at diagnosis, the German guideline recommends systemic steroid therapy (0.5–1 mg/kg body weight/day in prednisolone equivalents) ( **Box 4** ). In patients with mild-to-moderate left-sided colitis, however, the guideline specifically recommends budesonide MMX 9 mg/day 18 .

#### Translating the recommendations into evidence-based clinical practice

*Should budesonide MMX 9 mg/day be tried before systemic steroid therapy?*

The German guideline makes a specific recommendation for budesonide MMX in the case of patients with mild-to-moderate left-sided colitis in whom the recommended first-line treatments have been insufficient. Budesonide MMX is distinct from other systemic steroids because it is metabolized extensively on the first pass through the liver, which significantly reduces its systemic availability, thereby focusing its effects more locally within the gastrointestinal tract and minimizing broader side effects 48 49 . In everyday clinical practice, it is therefore advisable to consider budesonide MMX first in these patients, on a case-by-case basis, before moving on to systemic steroids. If a sufficient response is not achieved within 2–4 weeks depending on baseline disease activity, treatment escalation to systemic steroids should be considered.

*How to know if first-line mesalazine treatment is insufficient?*

The German guideline states that steroid therapy with budesonide MMX or systemic steroid treatment should be initiated if clinical symptoms worsen during treatment or if bleeding persists for more than two weeks 18 . More generally, according to STRIDE-II, physicians should consider changing treatment if there is no significant improvement within approximately four weeks when using optimized mesalazine 41 .

*When escalating to budesonide MMX 9 mg/day, should it be added to oral and/or rectal mesalazine, or should it be given as monotherapy?*

To our knowledge, only one study has looked at this specific question to date: a multicenter, prospective, real-world evidence cohort study conducted in Europe and Canada 50 . It found that adding budesonide MMX to optimized mesalazine treatment might be the best therapeutic approach to induce clinical remission compared to budesonide MMX monotherapy. Just over 60% of patients in the add-on treatment group achieved the primary endpoint of clinical benefit (improvement of ≥3 points in the UCDAI clinical subscore) compared to 33% in the monotherapy group, a substantial and statistically significant difference. Similar results were found for clinical remission (UCDAI clinical subscore ≤1) and symptom resolution (RB = 0, SF ≤1, and no urgency). Although only numerically different, higher percentages of patients who had budesonide added at least 14 days after mesalazine optimization achieved all three endpoints 50 , suggesting that at least two weeks of mesalazine therapy optimization might be beneficial before adding budesonide MMX.

### Maintenance of remission in mild-to-moderate UC

**Table:** **Table 5Box 5** . Maintenance of remission in mild-to-moderate UC

* Summarized recommendations (and related reference numbers from the guideline) ^‡^*	
3.13	Use mesalazine to maintain remission in patients who have responded to induction treatment with mesalazine or steroids.
3.14	The route of mesalazine administration in maintenance treatment should depend on the extent of disease. Proctitis and left-sided colitis should primarily be treated with rectal therapy.
3.15	Use combined oral/rectal mesalazine therapy as second-line treatment.
3.16c	Prefer mesalazine over sulfasalazine.
3.21	Do not use corticosteroids for maintenance of remission.
^‡^ Adapted from Kucharzik et al., 2024 18 .	

For maintaining remission in patients with mild-to-moderate UC ( **Box 5** ), the German guideline recommends mesalazine for all patients who have responded to induction treatment with mesalazine or steroids. In patients with proctitis or left-sided colitis, the guideline recommends monotherapy with rectal mesalazine as the first-line maintenance treatment. However, it acknowledges the challenges in adherence with rectal therapy and points to data indicating that patients with IBDs prefer not to be treated with suppositories or enemas 51 . For this reason, the guideline states that an alternative to rectal application is oral mesalazine formulations with a good release profile in the left-sided colon. In patients with more extensive UC, the guideline recommends monotherapy with oral mesalazine.

If rectal or oral monotherapy fails, the guideline recommends combination treatment with oral and rectal mesalazine. Because of its more favorable safety profile, mesalazine should be preferred over sulfasalazine. Corticosteroids should not be used for maintenance of remission.

#### Translating the recommendations into evidence-based clinical practice

*What role does therapy optimization play in maintenance treatment?*

In patients with left-sided colitis who indicate that they may have difficulty adhering to daily use of suppositories or enemas, it is reasonable to use combination treatment for maintenance, especially if combination treatment was already being used for induction. While the guideline states that monotherapy with oral mesalazine is an alternative in such cases, combination therapy allows for intermittent (twice per week) use of rectal mesalazine, which might be more acceptable to these patients. Above all, the choice of maintenance therapy should be thoroughly discussed with the patient and be based on patient preferences to ensure adherence 52 . Evidence shows that non-adherence to mesalazine therapy in quiescent UC is associated with a fivefold greater risk of recurrence 53 . Thus, selecting a regimen that the patient can consistently follow is crucial for long-term disease management.

*Is it beneficial to continue with mesalazine even after escalating to biologics/small molecules?*

To address this question, we conducted a systematic search of the literature published from 1995 onwards, which yielded 740 results. Title and abstract screening, performed independently by two authors, identified 18 potentially relevant records. After independent full-text review by two authors, 12 papers met the inclusion criteria and were included in the analysis. A detailed description of the search strategy, inclusion/exclusion criteria, results, and reasons for exclusion is provided in the Supplementary Appendix.

The search did not identify any RCTs specifically designed to assess whether continuing mesalazine is beneficial after escalation to biologics or small molecules in UC. The available evidence comes from retrospective analyses of clinical trials, population-based cohorts, and real-world settings. These studies showed no consistent evidence of short-term clinical benefit, such as improved rates of remission or mucosal healing, nor did they indicate an increased risk of harm, including any safety concerns, associated with concurrent mesalazine use. None of the included studies were powered or designed to investigate long-term outcomes such as colorectal cancer prevention.

Based on this evidence, we do not recommend a universal approach. Instead, we suggest that the decision to continue mesalazine after escalation should be individualized. In clinical practice, factors such as patient preference, disease history, previous response to mesalazine, and the potential (albeit inconsistently demonstrated) chemoprotective effect against colorectal cancer may support continuing treatment in selected patients. Given the favorable safety profile of mesalazine and the absence of evidence for harm in combination therapy, offering patients the option to continue mesalazine may be reasonable in cases where it aligns with patient needs and preferences.

**Table:** **Table 6Box 6** . Duration of maintenance therapy with mesalazine

* Summarized recommendations (and related reference numbers from the guideline) ^‡^*	
3.16d	Maintenance therapy should last at least two years.
3.18	Offer long-term treatment with mesalazine with a view towards cancer prevention
^‡^ Adapted from Kucharzik et al., 2024 18 .	

The German guideline recommends that maintenance therapy with mesalazine should last at least two years to reduce the risk of future relapses. It also recommends offering patients mesalazine treatment beyond two years due to its potential benefits in preventing colorectal cancer ( **Box 6** ).

#### Translating the recommendations into evidence-based clinical practice

*How long should maintenance treatment last, and why?*

The German guideline recommends long-term mesalazine (≥2 years) treatment not only to reduce the risk of relapse but also for its chemopreventive properties against colorectal cancer.

Reducing relapse risk : The German guideline recommends a minimum of two years for maintenance therapy with mesalazine. This recommendation is based on a controlled study showing that patients in remission for one to two years who continued on mesalazine therapy for an additional 12 months had a reduced incidence of relapses compared to those on placebo. Although this benefit was not observed in patients who had been in remission for more than two years, the statistical limitations of the study and lower dose of mesalazine (1.2 g/day) prevent definitive conclusions about the long-term benefits of maintenance therapy 18 .

Prevention of colorectal cancer : Long-term maintenance therapy with mesalazine may have the potential to prevent colorectal cancer, although the evidence remains weak. Nevertheless, due to the excellent safety profile of long-term mesalazine compared to biologics or small molecules, the German guideline recommends offering long-term treatment with mesalazine with a view towards cancer prevention. Chronic intestinal inflammation is a major risk factor for colorectal cancer in UC patients, and weak evidence indicates that ongoing mesalazine therapy is associated with reduced colorectal cancer risk 54 55 . This potential effect is thought to be mediated through the induction of apoptosis and decreased proliferation or modulation of carcinogenic gut microbiota and potentially of biofilm formation in colorectal mucosa 56 57 58 .

This putative preventive effect applies broadly to all UC patients 59 60 61 , except those with isolated proctitis 59 60 62 63 64 65 66 67 68 69 70 71 72 . The guideline contrasts this with the more limited evidence for a cancer protection effect of immunosuppressives like azathioprine and anti-TNF therapies in cancer prevention while highlighting their less favorable safety profile compared to mesalazine.

### Summary of dosage recommendations for maintenance of remission

**Table:** **Table 7Box 7** . Dosage recommendations for maintenance of remission

* Summarized recommendations (and related reference numbers from the guideline) ^‡^*	
(3.16a)	For oral mesalazine: use ≥2 g/day For rectal mesalazine alone: use ≥1 g/day For combined oral/rectal mesalazine therapy: oral 1.6–3 g/day plus rectal 1–4 g twice per week
(3.16b)	Prefer once-daily dosing
(3.17)	Consider *E. coli* strain Nissle 1917 as a potential alternative to mesalazine
^‡^ Adapted from Kucharzik et al., 2024 18 .	

The guideline specifies the following dosing regimens for mesalazine ( **Box 7** ): for oral administration, a minimum of 2 g/day is recommended; for rectal application alone, at least 1g/day is recommended. For those receiving combined oral/rectal therapy, the oral dose should range from 1.6–3 g/day with an additional 1–4 g of rectal mesalazine administered twice per week. The guideline also recommends opting for once-daily dosing to enhance patient adherence. While the non-pathogenic *E. coli* strain Nissle 1917 is recognized as a potential alternative to 5-aminosalicylates for maintaining remission in ulcerative colitis, the guideline notes that in clinical practice, mesalazine is more commonly used due to its larger body of supporting data and its potential effect in cancer prevention (see Recommendation 3.18) 18 .

## Overview of mesalazines and differences

Whether administered orally or rectally, mesalazine works by delivering the active anti-inflammatory component, mesalazine, directly to the site of inflammation in the colon. Regardless of the formulation or route of administration, systemic absorption is low, ensuring that the drug primarily acts locally in the gastrointestinal tract, which results in very few side effects and excellent tolerability 16 18 73 . Although the precise mode of action is unknown, mesalazine may reduce inflammation in the colon by inhibiting enzymes involved in the formation of inflammatory mediators like prostaglandin and leukotrienes 74 .

Mesalazine has been shown to have a safety profile comparable to placebo 16 23 24 . Its side effects are relatively mild and include headache, nausea, and abdominal pain, with rare but more serious effects like pancreatitis and nephritis. Importantly, higher doses of mesalazine do not appear to increase the risk of side effects 38 . In patients with UC, regular monitoring is important, with routine laboratory testing (e.g., creatinine, urea, AST, ALT, lipase, urinalysis) at least annually, even for asymptomatic patients. Several mesalazine formulations are available, each designed to optimize drug release at specific locations in the gastrointestinal tract. Although the German UC guideline notes comparable efficacy across products 18 , no head-to-head RCTs have been conducted to confirm this. Differences mainly concern their release profiles, influenced by pH dependency, prolonged-release mechanisms, and gastrointestinal factors such as transit time or inflammation. These distinctions, along with variations in excipients, may be relevant for patients with dietary preferences, comorbidities, or specific scheduling needs.

As of January 2025, a total of five mesalazine products, each with several formulations and doses, are available in the German market and approved for induction and maintenance treatment of UC. Listed in alphabetical order, these are: Asacol, Claversal, Mezavant, Pentasa, and Salofalk. The following section will provide an overview of these products, including their drug release profiles, dosage forms, strengths, as well as excipients and other ingredients.

### Drug-release profiles and kinetics of the different formulations

Each mesalazine formulation employs specific mechanisms to ensure appropriate drug release and therapeutic efficacy. These can be grouped into three categories ( Table 8 ):

**Table:** **Table 8** Drug-release profiles and kinetics of different mesalazine formulations (alphabetical order) based on information from the SmPCs.

		**Site of release**							
**Commercial product**	**pH release profile**	duodenum	jejunum	ileum	ascending colon	transverse colon	descending colon	sigmoid colon	rectum
**Asacol** enteric-coated tablets (0.4 g, 0.8 g) 75 76	pH-dependent			•	•	•	•	•	
**Asacol** modified-release tablets (1.6 g) 77	pH-dependent plus additional enzymatic activation (non-pH-dependent)			•	•	•	•	•	
**Claversal** micro-pellets (enteric-coated granules) (1.5 g) 78	pH-dependent			•	•	•	•	•	
**Claversal** enteric-coated tablets (0.5 g) 79	pH-dependent			•	•	•	•	•	
**Mezavant** enteric-coated, prolonged-release tablets (1.2 g) 80	pH-dependent				•	•	•	•	•
**Pentasa** prolonged-release granules (1 g, 2 g, 4 g) 81	Time-dependent (not pH-dependent)	•	•	•	•	•	•	•	•
**Pentasa** prolonged-release tablets (0.5 g, 1 g) 81 82	Time-dependent (not pH-dependent)	•	•	•	•	•	•	•	•
**Salofalk** Granu-Stix (prolonged-release granules) 83 (0.5 g, 1 g, 1.5 g, 3 g) 83	pH-dependent			•	•	•	•	•	•
**Salofalk** enteric-coated tablets (0.25 g, 0.5 g, 1 g) 84 85 86	pH-dependent			•	•	•	•	•	

*Eudragit coatings (pH-dependent).* Six mesalazine formulations rely exclusively on enteric coatings with Eudragit that dissolve at pH levels typically found in the ileum or colon: Asacol enteric-coated tablets 75 76 , Asacol modified release tablets 77 , Claversal micro-pellets (enteric-coated granules) 78 , Claversal enteric coated-tablets 79 , Salofalk Granu-Stix prolonged-release granules, and Salofalk enteric-coated tablets 83 84 85 86 . Their enteric coatings help protect the drugs from being released too early in the stomach and ensure that it reaches the intended location in the gastrointestinal tract. The site of release for Asacol is the terminal ileum and colon 75 , whereas for Claversal and Salofalk it is the mid to distal ileum and colon 79 84 . There are limited data on the extent to which these formulations are affected by other changes in gut conditions, such as diarrhea. One study from the Netherlands suggests that diarrhea had a significant effect on drug-release for Claversal, and Salofalk, and Asacol enteric-coated tablets 87 .

*Multi-matrix systems (MMX) (pH-dependent).* MMX systems, used for example in Mezavant enteric-coated, prolonged-release tablets, combine pH-dependent release with a slow-release matrix 80 88 .Specifically, Mezavant uses a combination of lipophilic and hydrophilic matrices 88 . This ensures extended drug release in the colon, allowing for a prolonged therapeutic effect and consistent drug delivery over time 80 88 . To the best of our knowledge, there are no data on whether diarrhea affects drug release.

*Ethylcellulose coating (time-dependent).* Two mesalazine formulations, Pentasa prolonged-release granules and Pentasa prolonged-release tablets, use a time-dependent, prolonged-release mechanism that is independent of pH 81 82 . It consists of ethylcellulose-coated microgranules that release mesalazine slowly throughout the gastrointestinal tract (duodenum to rectum), ensuring a more uniform distribution of the drug over a larger area of the intestines. The release of mesalazine in Pentasa is not significantly affected by diarrhea 87 .

### Excipients/other ingredients

There are large differences among the five mesalazine products in terms of their excipients and other ingredients ( Table 9 ). Understanding these differences is important for tailoring treatment to individual patient needs. For example, patients with lactose intolerance or sensitivities to sweeteners like aspartame may require specific formulations. Others on sodium-restricted diets may benefit from products with lower sodium content. Discussing these options with patients may improve satisfaction, adherence, and, ultimately, treatment outcomes.

**Table:** **Table 9** Excipients and other ingredients in oral mesalazine products currently available in Germany (alphabetical order) for the treatment of mild-to-moderate ulcerative colitis.

Brand	Formulation	Excipients and other ingredients (apart from mesalazine)
**Asacol**	Enteric-coated tablets 75 76	Lactose monohydrate (152.8 mg [800 mg] / 76.4 mg [400 mg]), carboxymethyl starch sodium (type A) (Ph.Eur.), talcum, povidone (25000), magnesium stearate (Ph.Eur.) [plant-based] (E 572), triethyl citrate, methacrylic acid-methyl methacrylate copolymer (1:2) (Ph.Eur.), iron (III) hydroxide oxide × H2O (E 172), iron (III) oxide (E 172), macrogol 6000
	Modified-release tablets 77	Magnesium stearate (E470B), methacrylic acid-methyl methacrylate copolymer (1:2), triethyl citrate, yellow iron oxide (E172), red iron oxide (E172), macrogol, microcrystalline cellulose, glycerol monostearate (40–55), hypromellose, corn starch, polysorbate 80, potassium dihydrogen phosphate, colloidal silicon dioxide, carboxymethyl starch sodium (type A)
**Claversal**	Micropellets (enteric-coated granules) 78	Microcrystalline cellulose, dispersible cellulose powder, citric acid, ascorbic acid, butylated hydroxyanisole, hypromellose, methacrylic acid-ethyl acrylate copolymer (1:1) MW: approx. 250,000 (Eudragit L 100–55); methacrylic acid-methyl methacrylate copolymer (1:2) MW: approx. 135,000 (Eudragit S 100), triethyl citrate, talcum, titanium dioxide, sucralose (Ph.Eur.), maltodextrin, tropical fruit flavor L-129243 Givaudan
	Enteric-coated tablets 79	Sodium carbonate, microcrystalline cellulose, highly dispersed silicone dioxide, glycine, povidone, croscarmellose sodium, calcium stearate, methacrylic acid-methyl methacrylate copolymer (1:1), methacrylic acid-methyl methacrylate copolymer (1:2), methacrylic acid-ethyl acrylate copolymer (1:1) dispersion 30%, talcum, triethyl citrate, titanium dioxide (E 171), iron (III) oxide (E 172), iron (III) hydroxide oxide (E 172), macrogol 6000
**Mezavant**	Enteric-coated, prolonged-release tablets 80	Tablet core: Carmellose sodium, carnauba wax, stearic acid (Ph.Eur.), silicone dioxide hydrate, sodium carboxymethyl starch (Type A) (Ph.Eur.), talcum, magnesium stearate (Ph. Eur.) [plant-based]; Film coating: Talcum (Ph.Eur.), methacrylic acid-methyl methacrylate copolymer (1:1) and (1:2) (Ph.Eur.), triethyl citrate, titanium dioxide (E171), iron (III) oxide (E172), macrogol 6000
**Pentasa**	Prolonged-release granules 81	Povidone, ethylcellulose
	Prolonged-release tablets 81 82	Povidone K30, ethylcellulose, magnesium stearate (Ph.Eur.), talcum, microcrystalline cellulose
**Salofalk**	Granu-Stix (prolonged- release granules) 83	Aspartame (E 951), carmellose sodium (Ph.Eur.), citric acid, highly dispersed silicone dioxide, hypromellose, magnesium stearate (Ph.Eur.) [plant-based], methacrylic acid-methyl methacrylate copolymer (1:1) (Ph.Eur.) (MW: approx. 135,000) (Eudragit L 100), methylcellulose, microcrystalline cellulose, polyacrylate dispersion 40% (Eudragit NE 40 D; contains 2% nonoxinol 100), povidone K 25, simethicone, sorbic acid (Ph.Eur.), talcum, titanium dioxide (E171), triethylcitrate, vanilla custard flavor (contains sucrose)
	Enteric-coated tablets 1g* 84 85 86	Microcrystalline cellulose, povidone K25, croscarmellose sodium, methacrylic acid-methyl methacrylate copolymer (1:1) (Ph.Eur.), methacrylic acid-methyl methacrylate copolymer (1:2) (Ph.Eur.), calcium stearate (Ph.Eur.) [plant-based], talcum, macrogol 6000, hypromellose, highly dispersed silicone dioxide, iron (III) hydroxide oxide × H2O, titanium dioxide (E 171)
* Composition of 250 mg and 500 mg tablets differs slightly		

### Available mesalazine dosage forms and strengths

A wide range of dosage forms and strengths of mesalazine exist, including tablets, granules, suppositories, rectal foam, and enemas/rectal suspensions. An overview of these is given in Table 10 (oral forms) and Table 11 (rectal forms).

**Table:** **Table 10** Currently available mesalazine oral dosage forms and strengths (alphabetical order).

Brand	Dosage form	Strengths	Licensed max. daily induction dose
Asacol	Enteric-coated tablets 75 76	0.4 g, 0.8 g	3.6 g, 4.8 g
	Modified-release tablets 77	1.6 g	4.8 g
Claversal	Micropellets (enteric-coated granules) 78	1.5 g	3 g
	Enteric-coated tablets 79	0.5 g	3 g
Mezavant	Enteric-coated, prolonged-release tablets 80	1.2 g	4.8 g
Pentasa	Prolonged-release granules 81	1 g, 2 g, 4 g	4 g
	Prolonged-release tablets 81 82	0.5 g, 1 g	4 g
Salofalk	Granu-Stix (prolonged-release granules) 83	0.5 g, 1 g, 1.5 g, 3 g	3 g
	Enteric-coated tablets 84 85 86	0.25 g, 0.5 g, 1 g	3 g

**Table:** **Table 11** Currently available mesalazine rectal dosage forms and strengths (alphabetical order).

Brand	Dosage form	Strengths	Licensed max. daily induction dose
Asacol	Suppositories 89	1 g	1 g
Claversal	Suppositories 90 91	0.25 g, 0.5 g, 1 g	1.0–1.5 g, 1.0–1.5 g, 1 g
	Rectal foam 92	1 g	2 g
	Enema 93	4 g/60 g	4 g
Pentasa	Suppositories 94	1 g	1 g
	Rectal suspension 95	1 g/100 ml	1 g
Salofalk	Suppositories 96 97 98	0.25 g, 0.5 g, 1 g	1.5 g, 1.5 g, 1 g
	Rectal foam 99	1 g	2 g
	Enema 100 101	2 g/30 ml, 4 g/60 ml	4 g

#### Considerations for patient-individualized mesalazine therapies

Given the variety of available products and formulations, several points should be considered when tailoring mesalazine treatment to individual patient needs. First, not all brands offer both oral and rectal formulations, limiting options for combination therapy. Unless absolutely necessary, we do not recommend mixing oral mesalazine formulations from different manufacturers or rectal mesalazine formulations from different manufacturers due to a lack of supporting evidence. Additionally, the release of most mesalazine products, except for one, is pH-dependent, which could potentially pose challenges for patients with irregular gut pH levels due to gastrointestinal conditions or medications, although this has not been systematically assessed in clinical trials or clinical practice. Such factors might also affect patients with frequent diarrhea, potentially altering the effectiveness of treatment for all formulations. To prevent potential allergic reactions, physicians should consider each product’s excipients and other ingredients, taking into account patient sensitivities and preferences. Optimizing therapy ideally involves selecting the highest optimized dose with the simplest regimen to improve adherence and treatment outcomes. Correct use of suppositories, foam, and enema should be explained in detail, and patient preferences should be considered.

To consolidate these points and other practical recommendations discussed throughout the paper, Table 12 provides a summary of the do’s and don’ts of mesalazine therapy in mild-to-moderate UC, offering clinicians a practical resource for translating the German UC guidelines into evidence-based daily practice.

**Table:** **Table 12** Do’s and don’ts of mesalazine therapy in mild-to-moderate UC.

DO’S	DON’TS
**DO** use mesalazine as first-line therapy in treatment of mild to moderative UC. **DO** use rectal mesalazine more often as combination therapy, not just for ulcerative proctitis. **DO** recommend once-daily dosing of mesalazine to improve adherence. **DO** always use rectal mesalazine with oral mesalazine for induction therapy when inflammation extends beyond the rectum. **DO** prefer rectal mesalazine therapy over rectal steroid therapy in proctitis. **DO** advise patients on the correct use of rectal mesalazine and to empty their bowel before treatment. **DO** instruct patients on how to insert suppositories correctly: warm them in your hands, briefly immerse them in water before insertion, and insert the blunt end first. **DO** instruct patients to administer enema and foam while lying on their left lateral side and remain resting for approximately 30 minutes. **DO** routine laboratory testing annually to check for side effects (e.g., creatinine, urea, AST, ALT, lipase, urinalysis). **DO** use mesalazine to maintain remission in patients who have responded to induction treatment with mesalazine or steroids.	**DON’T** overlook the excellent therapeutic effect of mesalazine in induction therapy for mild-to-moderate UC of any extent. **DON’T** underdose oral mesalazine for induction of remission. Use at least ≥3 g/day. **DON’T** split the mesalazine dose. Use it once daily. **DON’T** remove rectal mesalazine foam device too early – ensure it remains in place for at least 10–15 seconds in the anus to optimize its effectiveness. **DON’T** use creams or oils for inserting suppositories. **DON’T** mix oral mesalazine formulations from different manufacturers with each other or rectal mesalazine formulations from different manufacturers with each other due to a lack of evidence, unless absolutely necessary. **DON’T** stop mesalazine maintenance treatment too early. Use it for at least two years.

## Conclusion

Despite the advent of biologics and small molecule therapies, mesalazine remains the recommended first-line treatment of choice for patients with mild-to-moderate ulcerative colitis (UC) in all major UC treatment guidelines, including most recent update of the German Guideline on Ulcerative Colitis. These patients account for approximately 85% of all UC cases. In this paper, we reviewed the recommendations for mesalazine use in the German UC guideline and provided practical advice on how to translate them into clinical practice. The favorable effectiveness and safety profile of mesalazine makes it an optimal choice for rapidly inducing clinical remission, followed by long-lasting steroid-free clinical, endoscopic, and histologic remission. Optimizing mesalazine treatment offers several benefits, including the potential to achieve rapid clinical response, minimize the risk of recurrence, and avoid overtreatment and side effects associated with more aggressive therapies. Tailoring mesalazine therapy to individual patient needs through careful consideration of dosing regimens and formulations can improve treatment adherence and outcomes. Our systematic literature search evaluating whether mesalazine should be continued after escalation to biologics or small molecules found no conclusive evidence of short-term clinical benefit or harm, supporting a case-by-case decision process based on patient needs and preferences given mesalazine’s potential chemoprotective effect. It is important to recognize that the various mesalazine products are different from each other and therefore not interchangeable due to a lack of head-to-head comparisons. We described that each formulation has distinct drug-release profiles, and dosing strengths, which should be considered when prescribing and optimizing treatment. By understanding these differences, clinicians can ensure that patients with mild-to-moderate UC receive the most effective and tolerable therapy for their specific situation.

## Notes

Note This article was changed according to the following Erratum
**Correction**
In the above-mentioned article in Table 1 a bullet point in the rectum column for Salofalk Granu-Stix was included and “pH-dependent plus additional enzymatic activation (non-pH-dependent)” in the pH release profile column for Asacol 1600 mg modified-release tablets was added. Additionally in reference number 79 the link to the “Fachinformation (SmPC) Claversal 500 mg magensaftresistente Tabletten” was changed. The correct link in this case is as follows: https://www.fachinfo.de/fi/detail/021892/claversal-r-500-mg-tabletten?query=claversal%20500 .
This was corrected in the online version on March 13th, 2026.

## Supplementary Material

Supplementary Material Zusatzmaterial
