# Correction: From guidelines to evidence-based practice – A German perspective on mesalazine as first-line therapy for mild-to-moderate ulcerative colitis

**DOI:** 10.1055/a-2832-4215

**Published:** 2026-03-13

**Authors:** Elisabeth Schnoy, Axel Dignass, Matthew Gaskins, Torsten Kucharzik

**Affiliations:** 1 39694 Internal Medicine III, University Hospital Augsburg, Augsburg, Germany; 2 84491 Department of Medicine I, Goethe-University, Agaplesion Markus Hospital, Frankfurt, Germany; 3 Independent public health researcher, Madrid, Spain; 4 Department of Gastroenterology, Lüneburg Hospital, Lüneburg, Germany



In the above-mentioned article in Table 1 a bullet point in the rectum column for Salofalk Granu-Stix was included and “pH-dependent plus additional enzymatic activation (non-pH-dependent)” in the pH release profile column for Asacol 1600 mg modified-release tablets was added ( Table 1 ).

**Table:** **Table 1** Drug-release profiles and kinetics of different mesalazine formulations (alphabetical order) based on information from the SmPCs.

		**Site of release**							
**Commercial product**	**pH release profile**	duodenum	jejunum	ileum	ascending colon	transverse colon	descending colon	sigmoid colon	rectum
**Asacol** enteric-coated tablets (0.4 g, 0.8 g) [75, 76]	pH-dependent			•	•	•	•	•	
**Asacol** modified-release tablets (1.6 g) [77]	pH-dependent plus additional enzymatic activation (non-pH-dependent)			•	•	•	•	•	
**Claversal** micro-pellets (enteric-coated granules) (1.5 g) [78]	pH-dependent			•	•	•	•	•	
**Claversal** enteric-coated tablets (0.5 g) [79]	pH-dependent			•	•	•	•	•	
**Mezavant** enteric-coated, prolonged-release tablets (1.2 g) [80]	pH-dependent				•	•	•	•	•
**Pentasa** prolonged-release granules (1 g, 2 g, 4 g) [81]	Time-dependent (not pH-dependent)	•	•	•	•	•	•	•	•
**Pentasa** prolonged-release tablets (0.5 g, 1 g) [81, 82]	Time-dependent (not pH-dependent)	•	•	•	•	•	•	•	•
**Salofalk** Granu-Stix (prolonged-release granules) [83] (0.5 g, 1 g, 1.5 g, 3 g) [83]	pH-dependent			•	•	•	•	•	•
**Salofalk** enteric-coated tablets (0.25 g, 0.5 g, 1 g) [84, 85, 86]	pH-dependent			•	•	•	•	•	

Additionally in reference number 79 the link to the "Fachinformation (SmPC) Claversal 500 mg magensaftresistente Tabletten” was changed. The correct link in this case is as follows:

https://www.fachinfo.de/fi/detail/021892/claversal-r-500-mg-tabletten?query=claversal%20500 .

This was corrected in the online version on March 13th, 2026.

