# Supplementary material for: From guidelines to evidence-based practice – A German perspective on mesalazine as first-line therapy for mild-to-moderate ulcerative colitis
Source: Z Gastroenterol. 2025 Jun 16;63(10):1053–67. doi: 10.1055/a-2596-8934 (PMC12507481; doi:10.1055/a-2596-8934)
Supplement: Supplementary file 1 — Supplementary Material [file 10-1055-a-2596-8934_25969597.pdf]

Supplementary Appendix

Systematic search for research question: “Is it beneficial to continue mesalazine after escalating to biologics/small molecules in UC management?”

Search strategy and methods

A systematic literature search was conducted in PubMed on 29 January 2025 to investigate whether continuing mesalazine is beneficial after escalation to biologics or small molecules in patients with mild-to-moderate UC. The search included studies published from 1995 onwards, using keywords and MeSH terms related to UC, mesalazine, biologics, small molecules, and combination therapy.

We developed the search strategy based on the following PICO framework:

Research question: Is it beneficial to continue mesalazine after escalating to biologics/small molecules in UC management?

Population (P): Patients with UC, requiring escalation to biologics or small molecules

Intervention (I): Continuation of mesalazine (mesalamine, 5-ASA)

Comparator (C): Discontinuation of mesalazine after starting biologics or small molecules (or exclusive use of biologics/small molecules without mesalazine)

Outcome (O): Clinical outcomes such as maintenance of remission, mucosal healing, safety and tolerability, adherence, adverse events

The search string was as follows:

(ulcerative colitis[MeSH Terms] OR ulcerative colitis[Title/Abstract] OR UC[Title/Abstract]) AND (mesalazine[MeSH Terms] OR mesalazine[Title/Abstract] OR mesalamine[Title/Abstract] OR 5-aminosalicylic acid[Title/Abstract] OR 5aminosalicylic acid[Title/Abstract] OR 5-aminosalicylate*[Title/Abstract] OR 5aminosalicylate*[Title/Abstract] OR 5-ASA[Title/Abstract] OR 5ASA[Title/Abstract]) AND (biologics[MeSH Terms] OR biologic*[Title/Abstract] OR small molecule*[Title/Abstract] OR anti tumor necrosis factor[Title/Abstract] OR anti tumour necrosis factor[Title/Abstract] OR anti-TNF[Title/Abstract] OR infliximab[Title/Abstract] OR adalimumab[Title/Abstract] OR golimumab[Title/Abstract] OR Ustekinumab [Title/Abstract] OR vedolizumab[Title/Abstract] OR tofacitinib[Title/Abstract]) AND (English [Language] OR German[Language]) AND (1995[Date - Publication] : 3000[Date - Publication])

Predefined inclusion and exclusion criteria

Inclusion criteria:

- Peer-reviewed articles reporting clinical outcomes related to concurrent mesalazine use with biologics or small molecules in UC
- Included patients with mild-to-moderate UC (escalating therapy) or moderate-to-severe disease (initiating advanced therapies)
- Used retrospective, prospective, or observational designs, or analyzed clinical trial data
- Reported clinical outcomes such as remission, mucosal healing, safety, tolerability, or adherence

Exclusion criteria:

- Focused exclusively on ulcerative proctitis (UP), acute severe ulcerative colitis (ASUC), or pouchitis
- Were cost-effectiveness studies, case reports, or case series involving fewer than 20 participants
- Did not report clinical outcomes by concomitant mesalazine use
- Did not break down concomitant 5-ASA use by other therapies or did not clearly define treatment phases

Search results

The search yielded 747 records. Title and abstract screening was performed independently by two authors, leading to the exclusion of 729 records and the identification of 18 potentially relevant studies. Full-text screening was conducted independently by the same two authors, applying the predefined inclusion and exclusion criteria. Discrepancies were resolved by consensus. A total of 12 studies met the inclusion criteria and were included in the final analysis. The reasons for excluding the remaining six studies are listed at the end of this Supplementary Appendix.

Short summary of included studies

The 12 included studies were retrospective in nature and analyzed data from clinical trials, population-based cohorts, and real-world settings. Limitations included retrospective designs, a high proportion of intermittent mesalazine users, and limited adherence data. Further prospective, long-term studies are warranted to address this question definitively.

The review did not identify any RCTs that directly examined whether continuing mesalazine is beneficial after escalation to biologics or small molecules in UC. Of the 12 included studies, most (n=9) provided only indirect evidence, evaluating patients with moderate-to-severe disease who started induction treatment with an advanced therapy with or without concurrent mesalazine use, and thus did not represent the clinical situation of escalation

from mesalazine in the event of insufficient response. Three studies, however, provided direct evidence, including patients who started induction only on mesalazine, later escalated to advanced therapies, and were compared based on whether mesalazine was continued or discontinued.

Overall, none of the studies demonstrated clear evidence of benefit or harm from concurrent mesalazine use or continuing mesalazine after escalating to biologics or small molecules with regard to clinical outcomes such as remission, mucosal healing, or safety, although findings varied depending on the specific therapy and population studied. None of the studies were able to conduct a meaningful statistical analysis of the potential benefit of long-term 5-ASA use as a preventive agent against dysplasia and colorectal cancer.

Synthesis of findings

The 12 included studies differed in their designs, populations, and primary objectives, providing a mix of direct and indirect evidence relevant to our research question: whether continuing mesalazine is beneficial after escalation to biologics or small molecules in UC.

Studies providing direct evidence

Three studies – Ungaro et al. (2019), Bernstein et al. (2021), and Seo et al. (2024) – explicitly assessed the clinical outcomes of continuing versus discontinuing mesalazine after initiating biologic therapy. These studies generally included patients with mild-to-moderate UC, representing populations escalating therapy due to insufficient response to mesalazine or other conventional treatments. Collectively, they reported no clear benefit of continuing mesalazine in terms of remission, mucosal healing, hospitalization rates, or corticosteroid use, although they also did not identify any significant harms. Limitations included their retrospective designs, reliance on claims or prescription data, and the inability to assess adherence or the chemopreventive potential of mesalazine.

Studies providing indirect evidence

The remaining nine studies provided indirect evidence by analyzing mesalazine use based on data obtained in broader contexts, such as the pivotal clinical trials of advanced therapies (e.g., anti-TNFs, vedolizumab, or tofacitinib). These studies included populations with moderate-to-severe UC who had generally failed mesalazine therapy prior to enrolment, making their findings less directly applicable to patients with mild-to-moderate UC escalating from mesalazine due to insufficient response. The studies reported no significant associations between mesalazine use and efficacy outcomes such as remission, mucosal healing, or treatment persistence. Two exceptions were Nishida et al. (2025a) [1] and Nishida et al. (2025b) [2], who identified (a) a potential benefit of concomitant mesalazine use in reducing relapse rates in patients receiving lower doses of tofacitinib [1] and (b) a

possible protective effect of mesalazine against ustekinumab failure in subgroups of ulcerative colitis patients with prior exposure to only one advanced therapy [2].

Key limitations across these studies included short follow-up periods, post hoc or retrospective designs, and lack of data on adherence, dosing, and long-term chemopreventive effects.

Overall interpretation

Across the included studies, there was no consistent evidence of short-term benefit or harm associated with continuing mesalazine after escalation to biologics or small molecules. The direct evidence was limited by retrospective study designs, reliance on claims or prescription data that could not confirm adherence or reasons for mesalazine discontinuation, a lack of detailed clinical data to account for disease severity, and small sample sizes and/or short observation periods limiting the ability to detect potential chemopreventive effects of mesalazine. The indirect evidence predominantly included patients with moderate-to-severe UC who had failed mesalazine therapy before initiating biologics, making it less applicable to the clinical scenario of escalation from mesalazine in patients with mild-to-moderate UC. Despite the lack of clear short-term clinical benefits, the absence of harms and the potential for long-term chemopreventive effects suggest that continuation of mesalazine after therapy escalation may still be considered on a case-by-case basis, guided by patient-specific factors, such as patient needs and preferences, and clinical judgment.

Detailed summary of relevance of included studies to research question

Studies providing direct evidence

The studies in this category were explicitly designed to evaluate the benefits and harms of continuing versus stopping mesalazine after initiating biologics. They directly address our research question because their primary objective was to assess clinical outcomes in patients continuing versus discontinuing mesalazine following therapy escalation. While the studies did not explicitly define or restrict their populations to patients with mild-to-moderate UC, this can be reasonably inferred in cases where patients had been prescribed only mesalazine for induction therapy, as mesalazine is the standard first-line treatment for mild-to-moderate UC. The limitations summarized below include those reported in the publications as well as our interpretations based on the study methodologies, particularly in relation to their relevance to our research question.

1. **Bernstein, C. N. et al. (2021)** 'Continued 5ASA use after initiation of anti-TNF or immunomodulator confers no benefit in IBD: a population-based study', Alimentary Pharmacology & Therapeutics, 54(6), pp. 814-832. doi: 10.1111/apt.16518 [3].

   Design and methods: This retrospective cohort study used the population-based University of Manitoba IBD Epidemiologic Database to assess outcomes associated with continuing versus discontinuing 5-ASA after initiating anti-TNF therapy or immunomodulators. Outcomes assessed included hospitalization, surgery, corticosteroid initiation, colorectal cancer incidence, and drug-related adverse events. Data on medication use were based on prescription dispensations.

   Key findings: The study found no significant differences in adverse outcomes between the groups that continued versus discontinued 5-ASA after starting biologics or immunomodulators.

   Limitations:

   - Retrospective design limits causal inference
   - Reliance on prescription dispensation data, which may not reflect actual medication adherence
   - Absence of clinical chart review, preventing assessment of disease severity, activity, and reasons for 5-ASA discontinuation
   - High proportion of intermittent 5-ASA users, which may affect the interpretation of long-term outcomes (particularly potential chemoprotective effect of mesalazine)

2. **Seo, J. et al. (2024)** 'Continuing or stopping 5-aminosalicylates in patients with inflammatory bowel disease on anti-TNF therapy: A nationwide population-based study', Alimentary Pharmacology & Therapeutics, 60(3), pp. 389-400. doi: 10.1111/apt.18102 [4].

   Design and methods: This retrospective nationwide population-based study used Korean National Health Insurance claims data to compare the outcomes of continuing versus stopping 5-ASA within 90 days of initiating anti-TNF therapy in patients with IBD, including UC (n=2963). The primary outcome was adverse clinical events, defined as a composite of any new use of corticosteroids, hospitalization related to IBD, or intestinal surgery.

   Key findings: Discontinuation of 5-ASA was not associated with an increased risk of adverse clinical events (adjusted HR 1.01; 95% CI, 0.93-1.10) during a median of 4.3 years of follow-up.

Limitations:

- Retrospective design limits causal inference
- Absence of detailed clinical data, preventing assessment of disease severity, activity and reasons for 5-ASA discontinuation
- Reliance on prescription data, which may not accurately reflect medication adherence
- No information on the extent to which 5-ASA use was intermittent or continuous
- Study attempted to compare the incidence of colorectal cancer (CRC) between patients who stopped versus continued 5-ASA, but the very low number of CRC cases in both groups precluded meaningful statistical analysis

3. **Ungaro, R. C. et al. (2019)** 'Stopping 5-aminosalicylates in patients with ulcerative colitis starting biologic therapy does not increase the risk of adverse clinical outcomes: Analysis of two nationwide population-based cohorts', Gut, 68(6), pp. 977-984. doi: 10.1136/gutjnl-2018-317021 [5].

   Design and Methods: This study analyzed two national population-based cohorts (US: Truven MarketScan database; Denmark: Danish national health registries) to evaluate the outcomes of continuing versus stopping 5-ASA in patients with UC who escalated to anti-TNF therapy. The primary outcome was adverse clinical events, defined as a composite of new corticosteroid use, UC-related hospitalization, or intestinal surgery.

   Key Findings: Stopping 5-ASA was not associated with an increased risk of adverse clinical events in either cohort (US: adjusted HR 1.04; 95% CI, 0.90-1.21; Denmark: adjusted HR 1.09; 95% CI, 0.80-1.49). Median follow-up was approximately 9 months in the US cohort and 6 months in the Danish cohort, with similar durations between groups within each cohort.

   Limitations:

   - Retrospective design limits causal inference
   - Absence of detailed clinical data prevents assessment of disease severity, activity, and reasons for 5-ASA discontinuation
   - Reliance on prescription data, which may not accurately reflect medication adherence
   - No information on the extent to which 5-ASA use was intermittent or continuous
   - Study unable to fully evaluate potential chemoprotective benefit of 5-ASA due to very low number of events of advanced colorectal neoplasia in both cohorts, precluding meaningful statistical analysis

Studies providing indirect evidence

The studies in this category drew on data from trials or analyses designed to address broader research questions rather than explicitly aiming to evaluate the continuation versus discontinuation of mesalazine after therapy escalation. These studies analyzed mesalazine use as part of concurrent or combination therapy with biologics or small molecules. They predominantly included patients with moderate-to-severe UC, reflecting populations enrolled in pivotal clinical trials or observational studies of advanced therapies. While some of these trials included patients with an inadequate response to mesalazine, which partially aligns with our research question, this inclusion criterion was not consistently specified across studies. As a result, while the findings are informative, they provide only indirect evidence regarding our research question because they do not reliably reflect the clinical situation of patients who escalate from mesalazine due to insufficient response. The limitations summarized below include those reported in the publications as well as our interpretations of how the study designs relate to our research question.

1. **Ahuja, D. et al. (2024)** 'No Impact of Concomitant Medications on Efficacy and Safety of Biologics and Small Molecules for Ulcerative Colitis', Clinical Gastroenterology and Hepatology: The Official Clinical Practice Journal of the American Gastroenterological Association. doi: 10.1016/j.cgh.2024.08.040 [6].

   Design and Methods: This study conducted a pooled individual patient data analysis of 10 clinical trials of advanced therapies (e.g., infliximab, adalimumab, golimumab, vedolizumab, ustekinumab, and tofacitinib) in patients with moderate-to-severe UC. Baseline concomitant mesalazine use was included as a variable to assess its impact on treatment efficacy (clinical remission) and safety outcomes. The analysis employed a modified Poisson regression model to adjust for confounders.

   Key Findings: Baseline concomitant mesalazine use did not significantly affect the likelihood of clinical remission (ratio of risk ratio [RRR], 1.04; 95% CI, 0.78–1.39) or safety outcomes.

   Limitations:

   - Included only patients with moderate-to-severe UC, limiting its applicability to patients with mild-to-moderate UC escalating therapy
   - Analyzed baseline concomitant mesalazine use, without assessing ongoing or long-term use during induction or maintenance phases

2. **Balram, B. et al. (2021)** 'Concomitant 5-Aminosalicylate Therapy in Moderate-to-Severe Ulcerative Colitis Patients Escalated to Infliximab Is Not Beneficial'. Digestive Diseases and Sciences, 66(11), 3985–3992. doi: 10.1007/s10620-020-06704-6 [7].

   Design and methods: This single-center retrospective chart review analyzed 121 patients with moderate-to-severe UC who initiated infliximab therapy between January 2012 and

December 2017 at the University of Alberta, Canada. The study aimed to evaluate the impact of concomitant 5-ASA therapy on clinical outcomes. The primary outcome was clinical remission (partial Mayo score < 2) at six and 12 months. Secondary outcomes included endoscopic remission (Mayo endoscopic subscore < 2), deep remission (clinical and endoscopic remission), and adverse outcomes such as the need for rescue therapy, hospitalization, or colectomy. Logistic regression models were used to adjust for potential confounders.

Key findings: At six months, no significant difference in clinical remission was observed between patients receiving or not receiving concomitant 5-ASA (aOR 2.59, p = 0.07). At 12 months, patients on 5-ASA were significantly less likely to achieve endoscopic remission (aOR 0.08, p = 0.01) and deep remission (aOR 0.07, p = 0.02). Concomitant 5-ASA use did not significantly affect the need for rescue therapy, hospitalization, or colectomy. Patients on 5-ASA were more likely to receive concomitant immunomodulator therapy (73.3% vs. 54.1%, p = 0.03). The study suggests that 5-ASA does not provide additional benefit in achieving clinical, endoscopic, or deep remission in moderate-to-severe UC patients escalated to infliximab therapy. The authors conclude that discontinuation of 5-ASA in these patients may be appropriate.

Limitations:

- Included only patients with moderate-to-severe UC, limiting its applicability to patients with mild-to-moderate UC escalating therapy
- Single-center design limits generalizability, and small sample size reduces statistical power
- Included only patients with moderate-to-severe UC, limiting its applicability to patients with mild-to-moderate UC escalating therapy
- Retrospective design without a control group limits causal inference
- Reliance solely on logistic regression without advanced causal analysis techniques
- Long-term effects of discontinuing 5-ASA, such as its potential role in chemoprevention, were not assessed

3. **Choi, Y. I. et al. (2019)** ‘Comparison of outcomes of continuation/discontinuation of 5-aminosalicylic acid after initiation of anti-tumor necrosis factor-alpha therapy in patients with inflammatory bowel disease’, International Journal of Colorectal Disease, 34(10), pp. 1713–1721. doi: 10.1007/s00384-019-03368-1 [8].

Design and methods: This retrospective cohort study assessed 1300 patients with inflammatory bowel disease (IBD), including 63 with UC who had been prescribed biologics as induction and maintenance treatments at a single center. Patients were

divided into two groups based on whether they continued or discontinued 5-ASA. The primary outcome was event-free survival, defined as the time without disease exacerbation.

Key findings: For both UC and Crohn's disease, there were no significant differences in event-free survival between patients who continued versus discontinued 5-ASA.

Limitations:

- Unclear whether patients were exclusively on 5-ASA before initiating biologic therapy, limiting the ability to infer findings relevant to escalation from mesalazine
- Small sample size, particularly for subgroup analyses
- Single-center design, which may limit generalizability

4. **Nishida, Y. et al. (2025a)** 'Evaluating the effects of 5-aminosalicylic acid on tofacitinib treatment in ulcerative colitis', Journal of Gastroenterology and Hepatology. doi: 10.1111/jgh.16786 [1].

   Design and Methods: This retrospective cohort study used the Japanese Medical Data Vision database to assess the impact of concomitant 5-ASA use on relapse rates in UC patients treated with tofacitinib at doses of 5 mg or 10 mg twice daily. The primary outcome was clinical relapse, and multivariate Cox regression was used to adjust for potential confounders.

   Key Findings: Concomitant 5-ASA use significantly reduced the risk of relapse in the 5 mg BID group (adjusted HR, 0.47; 95% CI, 0.31-0.70). However, no benefit was observed with 5-ASA use in the 10 mg BID group (adjusted HR, 0.97; 95% CI, 0.71-1.32).

   Limitations:

   - Retrospective design limits causal inference
   - Findings may not generalize to populations using higher doses of tofacitinib or other treatment regimens
   - Lack of detailed clinical data prevents evaluation of disease severity, treatment adherence, and reasons for 5-ASA use or discontinuation
   - Study unable to assess the chemopreventive effects of 5-ASA due to the length of the follow-up period

5. **Nishida, Y. et al. (2025b)** 'Impact of 5-Aminosalicylic Acid on Ustekinumab in Inflammatory Bowel Disease: A Retrospective Medical Claims Analysis', Inflammatory Bowel Diseases. doi: 10.1093/ibd/izaf001 [2].

   Design and Methods: This retrospective cohort study analyzed data from the Medical Data Vision database to assess the impact of concomitant 5-ASA use on ustekinumab

treatment in patients with UC and Crohn's disease. The primary outcome was ustekinumab failure, defined as discontinuation of ustekinumab therapy. Kaplan-Meier survival analysis was used to evaluate cumulative ustekinumab continuation rates, and Cox proportional hazards models were employed to identify factors associated with ustekinumab failure. A post hoc subgroup analysis was conducted based on prior advanced therapy use.

Key Findings: A total of 1971 patients with Crohn's disease and 1284 patients with UC were included. Overall, concomitant 5-ASA use did not significantly affect ustekinumab failure rates in either UC or Crohn's disease. However, post hoc subgroup analyses suggested a protective effect of 5-ASA in advanced-therapy-naïve Crohn's disease patients and in UC patients who had prior exposure to only one advanced therapy (biologic or JAK inhibitor).

Limitations:

- Retrospective design limits causal inference
- Use of medical claims data prevents assessment of disease severity, treatment adherence, or reasons for ustekinumab failure
- Heterogeneous patient population with potential confounding due to prior therapies
- Potential for selection bias in the subgroup analysis; post hoc nature of subgroup findings warrants cautious interpretation
- Lack of endoscopic or clinical outcome data beyond ustekinumab continuation rates
- Study unable to assess the chemopreventive effects of 5-ASA due to the length of the follow-up period

6. **Seo, H. Il et al. (2014)** 'The effect of infliximab on patients with ulcerative colitis in Korea', Intestinal Research, 12(3), pp. 214-220. doi: 10.5217/ir.2014.12.3.214 [9].

   Design and Methods: This retrospective analysis evaluated 33 UC patients treated with infliximab at seven tertiary referral hospitals in Korea. The study investigated predictors of clinical remission and safety outcomes, including the impact of concurrent medication such as 5-ASA.

   Key Findings: Concurrent medication, including 5-ASA, was not significantly associated with achieving clinical remission or affecting safety outcomes in UC patients treated with infliximab.

Limitations:

- Retrospective design limits causal inference
- Small sample size limits statistical power and reliability of subgroup analyses
- Limited generalizability to broader UC populations due to the small and localized study cohort

7. **Singh, S. et al. (2018)** 'No Benefit of Concomitant 5-Aminosalicylates in Patients With Ulcerative Colitis Escalated to Biologic Therapy: Pooled Analysis of Individual Participant Data From Clinical Trials', The American Journal of Gastroenterology, 113(8), pp. 1197-1205. doi: 10.1038/s41395-018-0144-2 [10].

   Design and Methods: This pooled analysis assessed individual participant data from five clinical trials of infliximab and golimumab in patients with moderate-to-severe UC (n = 2183). The study examined the association between concomitant 5-ASA use and clinical outcomes, including clinical remission, clinical response, and mucosal healing. Multivariable logistic regression models were used to adjust for confounders.

   Key Findings: There was no significant association between concomitant 5-ASA use and clinical remission (adjusted OR, 0.67; 95% CI, 0.45-1.01; p=0.06), clinical response, or mucosal healing.

   Limitations:

   - Assessed only concomitant oral 5-ASA, excluding topical formulations, which may benefit patients with refractory proctitis
   - Post-hoc analysis of clinical trials, not a randomized trial specifically comparing biologic therapy with or without 5-ASA
   - Participants had already failed 5-ASA therapy before trial enrolment, meaning the study analyzed concurrent use of mesalazine during biologic treatment but did not assess true escalation from mesalazine to biologics due to an inadequate response
   - Study unable to evaluate potential chemopreventive effects of 5-ASA due to short follow-up periods

8. **Ungaro, R. C. et al. (2023)** 'Impact of Concomitant 5-Aminosalicylic Acid Therapy on Vedolizumab Efficacy and Safety in Inflammatory Bowel Disease: Post Hoc Analyses of Clinical Trial Data', Journal of Crohn's & Colitis, 17(12), pp. 1949-1961. doi: 10.1093/ecco-jcc/jjad113 [11].

   Design and Methods: This post hoc analysis evaluated clinical trial data from vedolizumab studies (GEMINI 1 and 2, VISIBLE 1 and 2) to assess the impact of 5-ASA co-treatment on efficacy and safety in patients with inflammatory bowel disease (IBD). Efficacy outcomes

included clinical and endoscopic remission rates at weeks 6 and 52. Safety outcomes were also analyzed.

Key Findings: No significant differences in clinical remission rates with vs. without 5-ASA co-treatment at week 6 (adjusted OR 0.77; 95% CI, 0.43-1.38) or week 52 (adjusted OR 1.14; 95% CI, 0.70-1.86). Safety outcomes were similar regardless of 5-ASA co-treatment.

Limitations:

- Post hoc analysis of RCT data limits causal inference
- Analysis limited to vedolizumab trials, reducing applicability to other advanced therapies
- Did not account for 5-ASA dose, duration, or adherence, which may influence outcomes
- Population was restricted to moderate-to-severe IBD patients, making findings less relevant to patients with mild-to-moderate UC escalating from mesalazine monotherapy

9. **Ylisaukko-oja, T. et al.** (2019) 'Characterization of inflammatory bowel disease management by vedolizumab and concomitant treatments in real-life clinical practice.', Biologicals: Journal of the International Association of Biological Standardization, 58(January), pp. 50–56. doi: 10.1016/j.biologicals.2019.01.007 [12].

Design and Methods: This retrospective chart review analyzed vedolizumab-treated inflammatory bowel disease (IBD) patients from 27 Finnish gastroenterology centers. Among other outcomes, the study evaluated the impact of baseline concomitant mesalazine use on treatment persistence and clinical outcomes at six months.

Key Findings: Concomitant mesalazine use did not significantly impact vedolizumab treatment persistence rates or clinical outcomes in vedolizumab-treated IBD patients over the six-month follow-up period.

Limitations:

- Retrospective design limits causal inference
- Short follow-up period (six months), limiting the ability to assess long-term outcomes
- No detailed information on mesalazine adherence or dose, which could influence findings
- Population was not stratified by disease severity (mild-to-moderate vs. moderate-to-severe UC)

Reasons for excluding individual studies from the full-text screening

- **Dai, C. et al. (2025).** 'The Effects of 5-Aminosalicylic Acid on Janus Kinase Inhibitor Treatment in Ulcerative Colitis.; J Gastroenterol Hepatol, 2025 Jan 11. doi: 10.1111/jgh.16867. Online ahead of print [13].
  → Reason for exclusion: Letter to the editor, does not meet inclusion criteria.
- **Hishinuma, K. et al. (2021)** 'Analysis of the Long-Term Prognosis in Japanese Patients with Ulcerative Colitis Treated with New Therapeutic Agents and the Correlation between Prognosis and Disease Susceptibility Loci', Inflammatory Intestinal Diseases, 6(3), pp. 154–164. doi: 10.1159/000518371 [14].
  → Reason for exclusion: No clinical outcomes reported according to concomitant mesalazine use.
- **Jang, E. J. et al. (2019)** 'A Real-World Analysis of Prescribing Patterns and Non-persistence of Anti-TNFα Therapy for Inflammatory Bowel Disease', Clinical Drug Investigation, 39(7), pp. 625–630. doi: 10.1007/s40261-019-00784-7 [15].
  → Reason for exclusion: No clinical outcomes reported according to concomitant mesalazine use.
- **Nielsen, K. R. et al. (2024)** 'Mortality of Patients With Inflammatory Bowel Disease in the Faroe Islands From 1966-2020', Inflammatory bowel diseases. doi: 10.1093/ibd/izae120 [16].
  → Reason for exclusion: Concomitant 5-ASA use not broken down according to other therapies (which included biologics, small molecules, and tacrolimus). For some outcomes, it was unclear whether the studies referred to induction treatment with 5-ASA followed by advanced therapy or concomitant use during maintenance therapy.
- **Rönnblom, A. and Karlbom, U. (2021)** 'Treatment and outcome of ulcerative colitis during the first 10 years after diagnosis in a prospectively followed population-based cohort', Scandinavian Journal of Gastroenterology, 56(4), pp. 403–409. doi: 10.1080/00365521.2021.1882553 [17].
  → Reason for exclusion: No clinical outcomes reported according to concomitant mesalazine use.
- **Wan, J. et al. (2023)** 'Geographical heterogeneity in the disease characteristics and management of patients with inflammatory bowel disease, the preliminary results of a Chinese database for IBD (CHASE-IBD)', Therapeutic Advances in Gastroenterology, 16, p. 17562848231210368. doi: 10.1177/17562848231210367 [18].
  → Reason for exclusion: No clinical outcomes reported according to concomitant mesalazine use.

References

[1] Nishida Y, Hosomi S, Fujimoto K, et al. Evaluating the effects of 5-aminosalicylic acid on tofacitinib treatment in ulcerative colitis. J Gastroenterol Hepatol. 2025;40(01):108-114. doi: 10.1111/jgh.16786

[2] Nishida Y, Hosomi S, Fujimoto K, et al. Impact of 5-Aminosalicylic Acid on Ustekinumab in Inflammatory Bowel Disease: A Retrospective Medical Claims Analysis. Inflamm Bowel Dis. 2025 Jan 11:izaf001. doi: 10.1093/ibd/izaf001. Epub ahead of print

[3] Bernstein CN, Tenakoon A, Singh H, et al. Continued 5ASA use after initiation of anti-TNF or immunomodulator confers no benefit in IBD: a population-based study. Aliment Pharmacol Ther. 2021;54(06):814-832. doi: 10.1111/apt.16518

[4] Seo J, Kim S, Hong SW, et al. Continuing or stopping 5-aminosalicylates in patients with inflammatory bowel disease on anti-TNF therapy: A nationwide population-based study. Aliment Pharmacol Ther. 2024;60(03):389-400. doi: 10.1111/apt.18102

[5] Ungaro RC, Limketkai BN, Jensen CB, et al. Stopping 5-aminosalicylates in patients with ulcerative colitis starting biologic therapy does not increase the risk of adverse clinical outcomes: analysis of two nationwide population-based cohorts. Gut. 2019;68(06):977-984. doi: 10.1136/gutjnl-2018-317021

[6] Ahuja D, Zou G, Solitano V, et al. No Impact of Concomitant Medications on Efficacy and Safety of Biologics and Small Molecules for Ulcerative Colitis. Clin Gastroenterol Hepatol. 2024 Oct 11:S1542-3565(24)00877-2. doi: 10.1016/j.cgh.2024.08.040. Epub ahead of print

[7] Balram B, Joshi H, Wong K, et al. Concomitant 5-Aminosalicylate Therapy in Moderate-to-Severe Ulcerative Colitis Patients Escalated to Infliximab Is Not Beneficial. Digestive Diseases and Sciences. 2020;66(11), 3985–3992

[8] Choi YI, Kim TJ, Park DK, et al. Comparison of outcomes of continuation/discontinuation of 5-aminosalicylic acid after initiation of anti-tumor necrosis factor-alpha therapy in patients with inflammatory bowel disease. Int J Colorectal Dis. 2019;34(10):1713-1721. doi: 10.1007/s00384-019-03368-1

[9] Seo HI, Park DI, Kim TO, et al. The Effect of Infliximab on Patients with Ulcerative Colitis in Korea. Intest Res. 2014;12(03):214-220. doi: 10.5217/ir.2014.12.3.214

[10] Singh S, Proudfoot JA, Dulai PS, et al. No Benefit of Concomitant 5-Aminosalicylates in Patients With Ulcerative Colitis Escalated to Biologic Therapy: Pooled Analysis of Individual Participant Data From Clinical Trials. Am J Gastroenterol. 2018;113(08):1197-1205. doi: 10.1038/s41395-018-0144-2

[11] Ungaro RC, Kadali H, Zhang W, et al. Impact of Concomitant 5-Aminosalicylic Acid Therapy on Vedolizumab Efficacy and Safety in Inflammatory Bowel Disease: Post Hoc Analyses of Clinical Trial Data. J Crohns Colitis. 2023;17(12):1949-1961. doi: 10.1093/ecco-jcc/jjad113

[12] Ylisaukko-Oja T, Torvinen S, Aaltonen J, et al. Characterization of inflammatory bowel disease management by vedolizumab and concomitant treatments in real-life clinical practice. Biol J Int Assoc Biol Stand. 2019;58:50-56. doi: 10.1016/j.biologicals.2019.01.007

[13] Dai, C and Huang Y-H. The Effects of 5-Aminosalicylic Acid on Janus Kinase Inhibitor Treatment in Ulcerative Colitis. J Gastroenterol Hepatol. 2025 Jan 11. doi: 10.1111/jgh.16867. Online ahead of print

[14] Hishinuma K, Moroi R, Okamoto D, et al. Analysis of the Long-Term Prognosis in Japanese Patients with Ulcerative Colitis Treated with New Therapeutic Agents and the Correlation between Prognosis and Disease Susceptibility Loci. Inflamm Intest Dis. 2021;6(03):154-164. doi: 10.1159/000518371

[15] Jang EJ, Ha JE, Im SG, et al. A Real-World Analysis of Prescribing Patterns and Non-persistence of Anti-TNFα Therapy for Inflammatory Bowel Disease. Clin Drug Investig. 2019;39(07):625-630. doi: 10.1007/s40261-019-00784-7

[16] Nielsen KR, Lophaven SN, Midjord J, et al. Mortality of Patients With Inflammatory Bowel Disease in the Faroe Islands From 1966-2020. Inflamm Bowel Dis. 2025 Apr 10;31(4):952-962. doi: 10.1093/ibd/izae120

[17] Rönnblom A, Karlbom U. Treatment and outcome of ulcerative colitis during the first 10 years after diagnosis in a prospectively followed population-based cohort. Scand J Gastroenterol. 2021;56(04):403-409. doi: 10.1080/00365521.2021.1882553

[18] Wan J, Shen J, Wu X, et al. Geographical heterogeneity in the disease characteristics and management of patients with inflammatory bowel disease, the preliminary results of a Chinese database for IBD (CHASE-IBD). Ther Adv Gastroenterol. 2023;16. doi: 10.1177/17562848231210367
